# Case Report: Lorlatinib combined with chemotherapy and anti-angiogenic therapy shows promising efficacy in an ALK gene fusion lung adenocarcinoma patient with iruplinalkib resistance

**DOI:** 10.3389/fphar.2026.1876652

**Published:** 2026-08-18

**Authors:** Mengqing Cao, Shumin Li, Linfeng Cao, Feng Tao, Na Li

**Affiliations:** Department of Respiratory Medicine, The Second Affiliated Hospital of Jiaxing University, Jiaxing, Zhejiang, China

**Keywords:** ALK-TKI, combination treatment strategy, EML4-ALK, iruplinalkib, lorlatinib, lung adenocarcinoma

## Abstract

**Background:**

The Echinoderm microtubule-associated protein-like 4 (EML4) - Anaplastic lymphoma kinase (ALK) gene fusion represents a significant driver mutation in non-small cell lung cancer (NSCLC). Among the over 20 identified EML4-ALK variant subtypes, the V5' variant occurs relatively infrequently, constituting merely 4% of cases. Currently, the clinicopathological features and prognostic implications associated with this particular variant remain poorly characterized in the literature.

**Case summary:**

We report the case of a 38-year-old man diagnosed with stage IV lung adenocarcinoma carrying the EML4-ALK V5' fusion variant, who received first-line treatment with iruplinalkib at a dose of 180 mg once daily. After 6.3 months of therapy, the patient developed a substantial malignant pleural effusion. Consequently, the treatment regimen was switched to lorlatinib 100 mg once daily combined with intrathoracic infusion chemotherapy. However, no significant improvement in the malignant pleural effusion was observed. We thereafter modified the therapeutic strategy to include lorlatinib-based targeted therapy combined with a dual-agent chemotherapy regimen consisting of cisplatin and pemetrexed, along with bevacizumab for anti-angiogenic therapy. Following this adjustment, the patient’s malignant pleural effusion showed marked remission.

**Conclusion:**

For patients with NSCLC harboring the EML4-ALK V5' fusion variant who have developed resistance to second-generation tyrosine kinase inhibitors (TKIs), switching to third-generation TKI lorlatinib in combination with chemotherapy and anti-angiogenic treatment may serve as a potential therapeutic option for overcoming acquired resistance and curbing rapid disease progression.

## Introduction

The anaplastic lymphoma kinase (ALK) gene fusion is one of the most critical oncogenic driver alterations in non-small cell lung cancer (NSCLC). Among these, the echinoderm microtubule-associated protein-like 4 (EML4)-ALK fusion gene, generated by an inverted fusion between the EML4 and ALK genes on human chromosome 2, is the most prevalent ALK fusion variant, with an estimated incidence of approximately 3%–7% in NSCLC patients (Soda, et al., 2007). Advances in molecular detection technology have led to the identification of more than 20 distinct EML4-ALK variant subtypes (Lin, et al., 2018; Schneider, et al., 2023). Emerging evidence suggests that different ALK fusion variants may differentially influence tumor biological behavior, baseline sensitivity to ALK-tyrosine kinase inhibitors (TKIs), and mechanisms of acquired resistance (Woo, et al., 2017). In contrast to the more commonly observed V1 and V3 variants, the V5' variant, resulting from the fusion of EML4 exon 18 to ALK exon 20 (E18; A20), exhibits a low frequency, accounting for only 4% of EML4-ALK fusion-positive NSCLC cases. Consequently, its clinicopathological characteristics and prognostic data remain poorly defined (Yoshida, et al., 2016). For patients harboring EML4-ALK V5' fusion variants, second-generation ALK inhibitors, including alectinib and iruplinalkib, are typically employed as first-line therapy. Nevertheless, clinical management following resistance to second-generation TKIs drug poses a substantial therapeutic challenge. Herein, we present a comprehensive account of the diagnostic workup and treatment course of a patient with advanced lung adenocarcinoma carrying this rare EML4-ALK V5' fusion variant, with a particular focus on the therapeutic decisions made following disease progression on iruplinalkib.

## Case presentation

**FIGURE 1:**
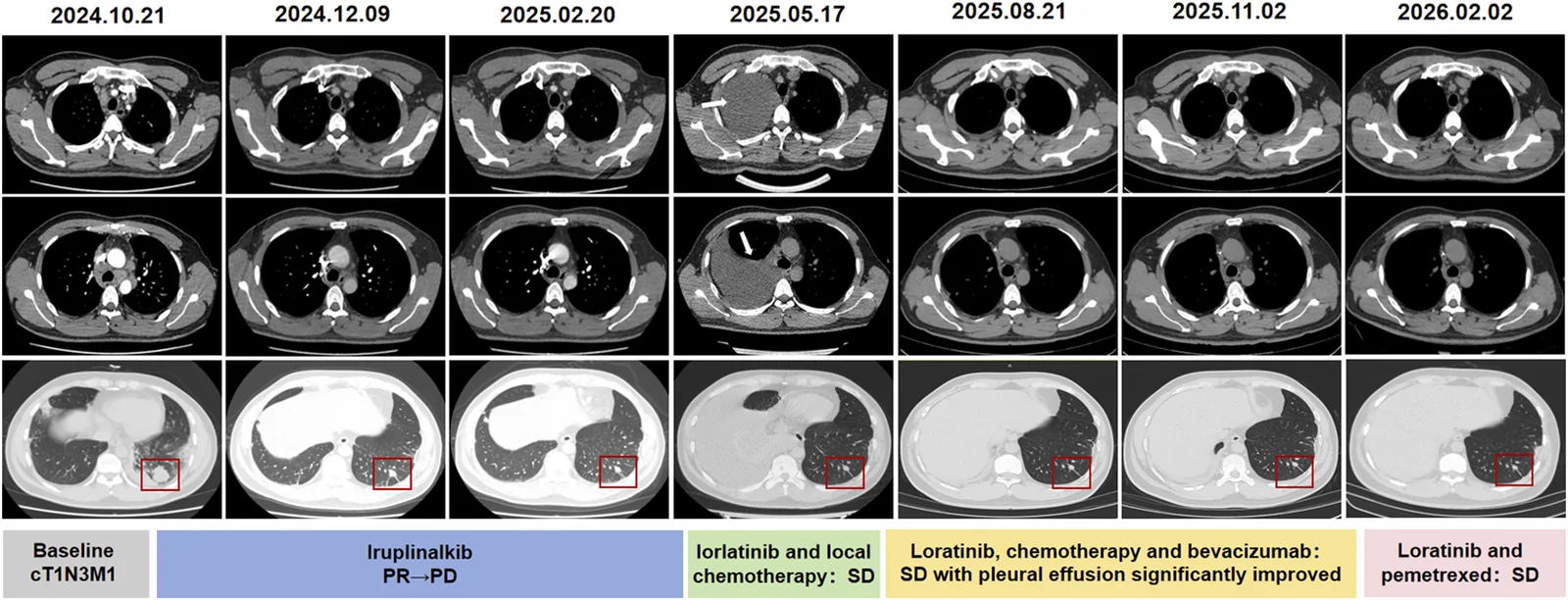
The patients’ therapeutic timeline. Red label marks the target lesion. White arrow points to pleural effusion.

**FIGURE 2:**
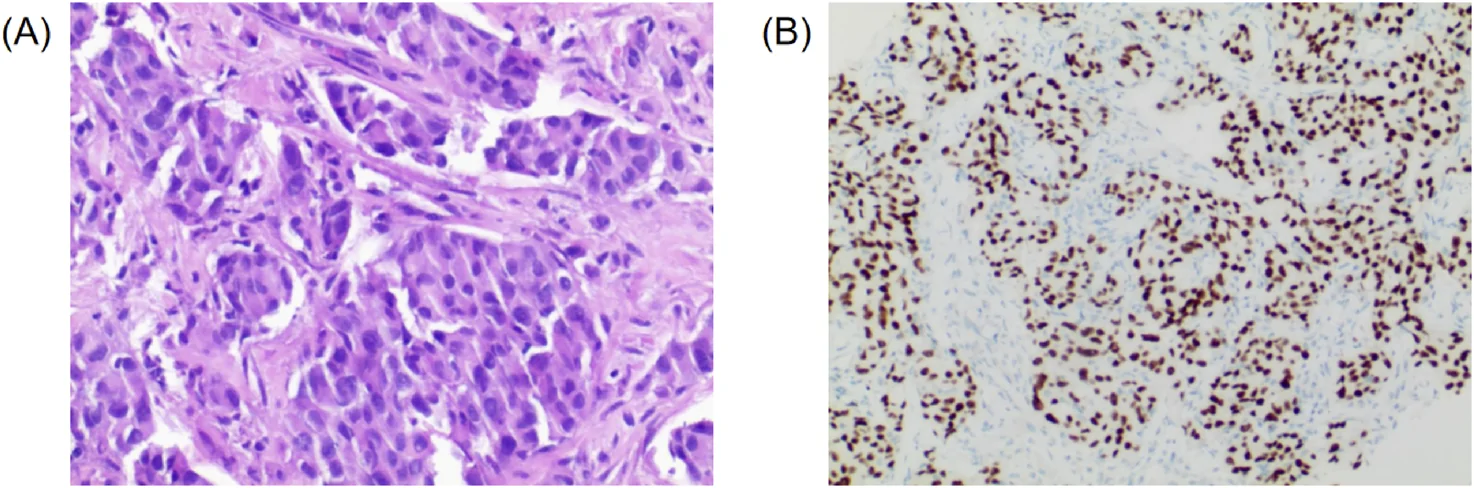
Pathological biopsy of right supraclavicular lymph node on 24 October 2024. **(A)** Hematoxylin and Eosin Staining (×200). **(B)** Thyroid Transcription Factor 1 expression.

A 38-year-old Chinese man with a 1-year history of type 2 diabetes mellitus presented with chest pain on 20 October 2024. Contrast-enhanced computed tomography (CECT) of the chest revealed a 27 mm × 28 mm mass in the lower lobe of the left lung, accompanied by enlarged mediastinal lymph nodes (stations 2R, 4L and 4R per IASLC classification), a small pleural effusion, emboli in both lower pulmonary arteries and multiple areas of abnormal bone mineral density in the thoracic and lumbar spine (Figure 1). Color Doppler ultrasonography of the cervical lymph nodes identified enlarged right supraclavicular nodes, suggestive of lung cancer with possible lymphatic metastases. Immunohistochemical (IHC) analysis from the right supraclavicular lymph node yielded the following results: CK(+), TTF-1(+), P40 (−), Ki-67(+, local index approximately20%), INSM1 (−), CDX2 (−), CHGA (−), NapsinA(+), CD34 (−), S-100(partial+), PAX-8 (−), P63 (−). These findings were consistent with a diagnosis of lung adenocarcinoma with lymph node involvement (Figure 2). The preliminary clinical diagnosis was adenocarcinoma of the left lower lobe, cT1N3M1, Stage IV (AJCC 8th edition staging) with supraclavicular lymph nodes and multiple bone metastases, and an Eastern Cooperative Oncology Group (ECOG) performance status score of 0. Programmed death-ligand 1 (PD-L1) expression testing revealed a tumor proportion score (TPS) of 20%. Next-generation sequencing (NGS) performed on right supraclavicular lymph node puncture specimen identified an EML4 exon 18 -ALK exon 20 fusion (allele fraction: 12.90%), along with MDM2 amplification (copy number: 3.1) and KIT amplification (copy number: 2.7). These molecular findings established the basis for subsequent precision targeted therapy.

### Stage 1 (first-line TKI treatment and initial response)

Based on the presence of the EML4-ALK V5' variation, the patient initiated treatment with second-generation ALK-TKI iruplinalkib at a dose of 180 mg once daily (60 mg once daily during the first week), along with denosumab 120 mg every 4 weeks for bone protection on 8 November 2024. At the time of initial presentation on 20 October 2024, laboratory testing revealed a total cholesterol level of 5.34 mmol/L and triglyceride level of 4.06 mmol/L, indicative of hypercholesterolemia, although the patient reported no prior history of hyperlipidemia. Given that ALK-TKI therapy may exacerbate preexisting hyperlipidemia, rosuvastatin 5 mg daily was initiated on 8 November 2024, for lipid management. Additionally, due to the presence of pulmonary embolism, rivaroxaban was administered at a dose of 20 mg once daily (following a 3-week loading regimen of 15 mg twice daily) from 8 November 2024. Anticoagulation therapy was subsequently discontinued because the patient developed recurrent epistaxis (CTCAE grade 2), after a total treatment duration of 6 months. Re-examination on 9 December 2024 revealed substantial reduction in the pulmonary mass, node metastases and thrombotic burden, confirming the initial therapeutic efficacy of iruplinalkib against this V5' variant (Figure 1). The efficacy evaluation was partial response (PR) attending to Response Evaluation Criteria in Solid Tumours (RECIST), and the progression-free survival (PFS) was 6.3 months (8 November 2024 - 17 May 2025).

### Stage 2 (acquired drug resistance and treatment strategy adjustment)

**FIGURE 3:**
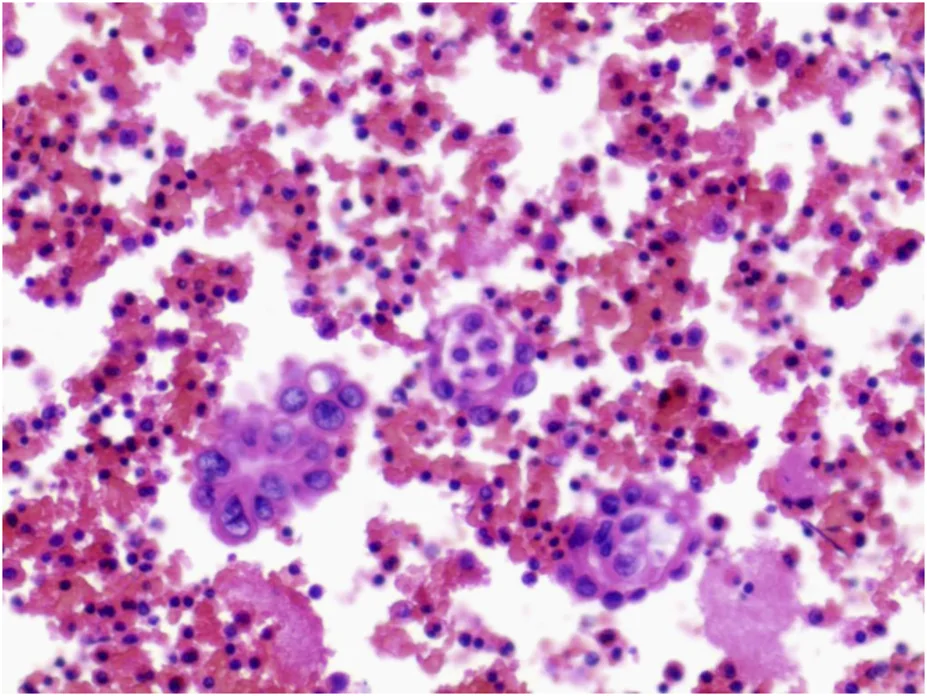
Pleural effusion cytopathology on 18 May 2025 (×200).

On 17 May 2025, the patient was hospitalized with dyspnea. Chest computed tomography (CT) demonstrated reduction in the left lower lung mass but a large right-sided pleural effusion (Figure 1). Malignant cells were identified in the pleural fluid (Figure 3), confirming disease progression (PD) per RECIST. Pleural effusion tumor markers revealed markedly elevated carcinoembryonic antigen (117.7 ng/mL) and neuron-specific enolase (>370 ng/mL). Because patients with ALK fusion may acquire secondary ALK mutations after second-generation TKI exposure and given the patient’s refusal of repeat NGS testing due to cost, we switched therapy to lorlatinib 100 mg once daily on 20 May 2025, a third-generation ALK-TKI with a broader spectrum of resistance mutations. Denosumab 120 mg every 4 weeks was maintained for bone support. To expedite control of the pleural effusion, two sessions of intrapleural instillation with cisplatin 30 mg plus interleukin-2 2 million IU were performed starting 27 May 2025. The patient experienced transient relief of dyspnea, which was followed by rapid symptom recurrence.

### Stage 3 (combination therapy)

**FIGURE 4:**
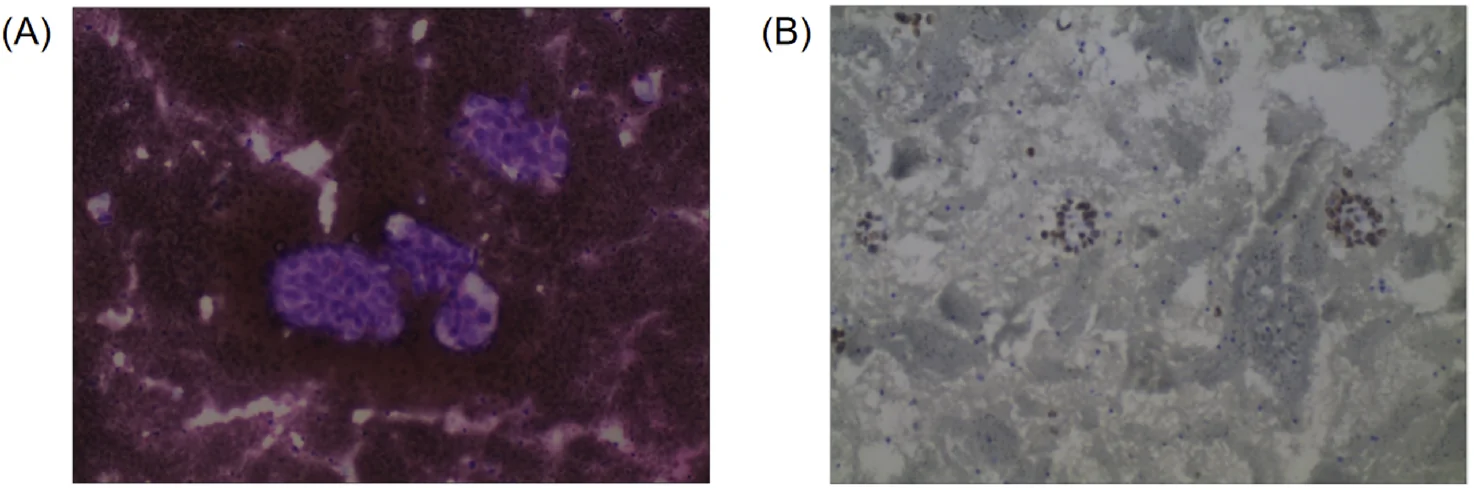
Cytopathological and immunohistochemical analysis of right pleural effusion on 4 July 2025. **(A)** Hematoxylin and Eosin Staining (×200). **(B)** Thyroid Transcription Factor 1 expression.

On 4 July 2025, the patient was hospitalized again due to dyspnea. Chest color Doppler ultrasonography revealed a large right-sided pleural effusion, prompting therapeutic thoracentesis. IHC analysis of the pleural fluid showed: TTF-1(+), NapsinA(+), P40(−), CK5/6 (−), Ki-67(+, local index approximately 5%), CK(+), indicating lung adenocarcinoma with pleural metastasis (Figure 4). Meanwhile, imaging evaluation of other disease sites indicated stable disease control (Figure 1). The overall response to targeted therapy combined with intrathoracic infusion chemotherapy was assessed as stable disease (SD) per RECIST. Pleural fluid tumor markers showed a decline in carcinoembryonic antigen to 11.7 ng/mL and neuron-specific enolase to 80.18 ng/mL. Given the marked reduction in pleural fluid tumor marker levels, we hypothesized that a subset of tumor cells producing these markers remained sensitive to the combination of lorlatinib and local therapy. However, the persistent volume of malignant pleural effusion suggested the presence of a residual tumor cell population on the pleura that had not been effectively controlled, potentially harboring acquired resistance mutations. Considering that the malignant effusion substantially impaired the patient’s quality of life, we decided to modify the treatment strategy. Given the observed decline in pleural fluid tumor marker levels following prior therapy, we recommended continuation of lorlatinib. In the absence of medical talc availability in China, the lack of randomized controlled trial evidence for intrapleural anti-angiogenic therapy, and the associated high costs, we adopted a chemo-targeted combination approach. The revised regimen consisted of lorlatinib 100 mg once daily, dual-agent chemotherapy with cisplatin and pemetrexed, bevacizumab for anti-angiogenic therapy, and denosumab for bone protection.

Starting 10 July 2025, the patient received pemetrexed 0.8 g on day 1, cisplatin 40 mg on days 1–3,and bevacizumab 0.6 g on day 1, all via intravenous drip. On July 30, follow-up chest ultrasonography demonstrated a marked reduction in pleural effusion, indicating rapid therapeutic efficacy. The same regimen was continued on August 1. Notably, after this treatment, the patient experienced recurrent mild hemoptysis (CTCAE grade 2). On August 21, the patient was readmitted for restaging evaluation, which showed further reduction in right pleural effusion and stability of other lesions (Figure 1). Despite the label recommendation against dose reduction of bevacizumab, a 2024 case report documented a hepatocellular carcinoma patient who experienced variceal bleeding on atezolizumab plus bevacizumab. After the bevacizumab dose was halved while atezolizumab was continued, the patient had no recurrent variceal bleeding (Yeom, K. M. et al., 2024). Given the suspicion of bevacizumab-related bleeding, we recommended dose reduction of bevacizumab. Starting from August 22, the patient received pemetrexed 0.8 g on day 1, cisplatin 30 mg on day 1–3, and bevacizumab 0.4 g on day 1. Following this dose adjustment, the hemoptysis resolved. The patient subsequently received the same anti-tumor regimen on September 12, October 9, and 3 November 2025. On November 16, the patient complained of frequent epistaxis (CTCAE grade 2). Considering bevacizumab related bleeding adverse reactions, we recommended discontinuation of bevacizumab, with continuation of lorlatinib combined with pemetrexed maintenance monotherapy. After bevacizumab cessation, the epistaxis resolved. On 2 February 2026, chest CT review demonstrated stable lung mass and lymph node lesions (Figure 1).

## Discussion

The core value of this case lies in its clear background of EML4-ALK V5' fusion variant, which provides a practical basis for exploring the clinical management of this rare subtype after iruplinalkib resistance.

### Driver gene and clinical characteristic

The V5' variant originated from the inversion of the short arm of chromosome 2, causing the fusion of EML4 exon 18 to ALK exon 20 (Zhang et al., 2021). The phase III INSPIRE study (NCT04632758) was designed to evaluate the efficacy and safety of iruplinalkib versus crizotinib in patients with ALK-positive advanced NSCLC who had not previously received systemic treatment, with the primary endpoint of PFS assessed by an independent review center. Updated data presented at the 2024 ESMO congress report a median PFS of 36.80 months and 14.55 months for the iruplinalkib and crizotinib group, respectively (van der Wel and de Langen, 2025). In this case, the patient achieved a PFS of 6.3 months using second-generation TKI iruplinalkib, confirming that the EML4-ALK V5' variant is sensitive to second-generation TKI. Previous studies have demonstrated that distinct EML4-ALK variants subtypes differentially modulate TKI sensitivity by affecting fusion protein stability, dimerization capacity, and downstream signaling pathway activation efficiency (Shaw et al., 2013). Due to the relatively low prevalence of the V5' variant, its specific resistance profile and prognostic implications remain to be elucidated. A pooled analysis reported a thrombosis risk of approximately 30% among patients with ALK fusion-positive tumors, significantly higher than the 12% observed in EGFR-mutant cases and 14% in wild-type cases (Liu et al., 2021). Subgroup analysis further revealed that ALK fusion-positive patients who developed thromboembolism had significantly shorter PFS following first-line TKI therapy than those without thromboembolic events. The markedly shorter PFS observed in our patient compared with the INSPIRE trial population may therefore be attributable to both the V5' variant subtype and the presence of thromboembolism. These findings carry important implications for the clinical management of similar patients. Future strategies may consider the earlier adoption of more intensive combination regimens or the incorporation of circulating tumor DNA (ctDNA) monitoring during TKI therapy to guide timely treatment adjustments.

### Mechanism of drug resistance and subsequent drug selection

Acquired resistance to ALK-TKIs remains an inevitable clinical challenge. Mechanisms of drug resistance can be divided into two categories: ALK-dependent mechanisms such as secondary ALK kinase domain mutations, ALK copy number increases and ALK-independent mechanisms such as bypass activation, histological type transformation (Fukui, et al., 2022). In a study on the mechanism of crizotinib resistance, researchers analyzed tumor samples from 18 crizotinib-resistant patients and found that significant amplification of KIT can activate bypass pathways to restart downstream cancer-promoting signaling pathways, which is one of the key reasons for drug resistance (Katayama et al., 2012). Mechanistically, the resistance observed in our patient may be attributable to bypass activation driven by KIT amplification. However, as post-progression molecular profiling was not performed at the patient’s request, this interpretation remains speculative. Compared with oligopoly progression, extensive progression usually means more complex drug resistance mechanisms, stronger tumor heterogeneity, and relatively worse prognosis. Notably, secondary ALK kinase domain mutations account for over 50% of treatment failures in patients receiving second-generation ALK-TKIs, constituting the predominant mechanism of acquired resistance (Schneider, et al., 2023). Following first-line TKI failure, repeat NGS is generally recommended to elucidate the resistance mechanism; nevertheless, our patient declined this testing owing to financial constraints. Different first-line TKIs and baseline variant subtypes are associated with distinct resistance mutation spectra. For instance, the V3 variant exhibits a higher propensity for acquiring the G1202R mutations following second-generation TKI therapy (L. Horn, et al., 2019). Although data on the specific resistance profile of the V5' variant remain extremely limited, we selected lorlatinib as the second-line treatment option due to its potent activity against a broad spectrum of common resistance mutations. However, 1 month after switching to lorlatinib, malignant pleural effusion remained poor controlled, severely compromising the patient’s quality of life and prompting us to pursue a more intensive therapeutic strategy.

### The role of joint strategy in managing TKI resistance

The patient developed right-side malignant pleural effusion, whereas the primary tumor was located in the left lower lobe. The underlying mechanism may be related to lymphatic metastasis: enlargement of the 2R and 4R lymph nodes block the backflow of lymph fluid from the right pleura, increasing the pressure in the pleural lymphatic network, and some lymph fluid may carry tumor cells countercurrent and spread to the right pleura. An *in vitro* study explored the efficacy of ALK-TKI combined with chemotherapy in ALK fusion-positive NSCLC, and selected alectinib as the representative of ALK-TKI, and cisplatin plus pemetrexed as the representative of chemotherapy drugs (Luukkainen and Koivunen, 2024). In colony formation assays simulating long-term treatment, aletinib combined with cisplatin demonstrated a synergistic effect in ALK fusion-positive cell lines, effectively inhibiting long - term tumor cell survival. In short-term activity assays, the combination of alectinib and pemetrexed showed a synergistic effect. The study concluded that the combination of TKI and chemotherapy may help overcome primary TKI resistance, which provides a solid biological foundation for the clinical application of targeted drug combination chemotherapy. For patients with malignant pleural effusion, combining thoracic infusion or systemic chemotherapy with sequential TKI therapy represents an active management strategy. This multi - modality approach aims to achieve tumor inhibition through complementary mechanisms and provide rapid symptomatic relief. Therefore, we cannot determine whether the benefit was primarily from lorlatinib, from chemotherapy plus bevacizumab, or from the combination as a whole.

### TKI adverse drug reaction management

TKI-related adverse reactions require early identification and active intervention to ensure treatment continuity and preserve patient quality of life. Common adverse effects of lorlatinib include hyperlipidemia, nervous system toxicity and abnormal liver function. In this case, given that the patient’s baseline serum triglycerides were 4.06 mmol/L, we initiated rosuvastatin for lipid management and closely monitored lipid profiles throughout ALK-TKI therapy, with particular attention to the differential effects of iruplinalkib and lorlatinib on lipid metabolism. One month after initiating iruplinalkib, the patient developed Grade 3 hyperlipidemia with a serum triglyceride level of 9.41 mmol/L. Notably, on subsequent follow-up, his total cholesterol level dropped to 5.48 mmol/L and his triglyceride level declined to 3.17 mmol/L without any adjustment to the lipid-lowering regimen. In contrast, half a month after transitioning to lorlatinib, the patient developed Grade 4 hyperlipidemia, with serum triglyceride reaching 19.41 mmol/L. During rosuvastatin treatment, the hyperlipidemia dropped to Grade 3. Given that lipid levels remained poor controlled, we sequentially added fenofibrate and ezetimibe to the lipid-lowering regimen. By 29 December 2025, the patient’s total cholesterol has decreased to 7.35 mmol/L and triglyceride to 2.40 mmol/L, indicating resolution of hyperlipidemia to a mild degree. The Phase III CROWN study reported that hypercholesterolemia and hypertriglycerides occurred in 72% and 66% of patients receiving lorlatinib, respectively, with severe elevations observed in 25% and 21% of patients, respectively (Liu et al., 2025). These findings underscore the importance of regular laboratory monitoring and timely adjustment of lipid-lowering therapy to ensure the safe and sustained delivery of ALK-TKI treatment.

## Conclusion

This case confirms sensitivity of the EML4-ALK V5' variant to ALK-TKIs, though the response duration requires confirmation in larger studies. For patients progressing on second-generation TKIs, transitioning to the third generation agent lorlatinib in combination with chemotherapy and antiangiogenic therapy. Future prospective research and translational medicine exploration are needed to reveal the specific resistance mechanisms of rare ALK variants such as V5', thereby informing more rational sequencing of TKIs and optimization of combination regimens, with the ultimate goal of improving long term outcomes for all patients with ALK positive NSCLC.

## Data Availability

The raw data supporting the conclusions of this article will be made available by the authors, without undue reservation.
